# Hypopituitarism other than sellar and parasellar tumors or traumatic brain injury assessed in a tertiary hospital

**DOI:** 10.12669/pjms.35.4.174

**Published:** 2019

**Authors:** Sarwar Malik, Zareen Kiran, Muhammad Owais Rashid, Minaz Mawani, Asma Gulab, Muhammad Qamar Masood, Najmul Islam

**Affiliations:** 1 Dr. Sarwar Malik, FCPS (Medicine), FCPS (Endocrinology). Department of Medicine, Federal Govt. Polyclinic Hospital, Islamabad, Pakistan; 2 Dr. Zareen Kiran, FCPS (Medicine), MRCP (UK), FCPS (Endocrinology). Department of Medicine, Aga Khan University Hospital, Karachi, Pakistan; 3 Dr. Muhammad Owais Rashid, FCPS (Medicine). Department of Medicine, Aga Khan University Hospital, Karachi, Pakistan; 4 Ms. Minaz Mawani, MSc (Epidemiology and Biostatistics). Department of Medicine, Aga Khan University Hospital, Karachi, Pakistan; 5 Dr. Asma Gulab, MBBS. Aga Khan Medical College, Aga Khan University Hospital, Karachi, Pakistan; 6 Dr. Muhammad Qamar Masood, Diplomate American Board of Endocrinology, Department of Medicine, Aga Khan University Hospital, Karachi, Pakistan; 7 Dr. Najmul Islam, FRCP. Department of Medicine, Aga Khan University Hospital, Karachi, Pakistan

**Keywords:** Empty sella syndrome, Hypopituitarism, Panhypopituitarism, Sheehan’s syndrome

## Abstract

**Objective:**

Data regarding the etiology, clinical and biochemical patterns in hypopituitarism is scant for Pakistan. We describe the characteristics of patients with hypopituitarism other than sellar and parasellar tumors or traumatic brain injury from a tertiary care center in Pakistan.

**Methods:**

We conducted a retrospective descriptive study in the Aga Khan University Hospital, Karachi, Pakistan. We studied all patients presenting with hypopituitarism, between January 2004 and December 2013. Clinical, hormonal and imaging data pertinent to the study was collected according to inclusion criteria.

**Results:**

Forty-two patients presented to the endocrinology clinics at the Aga Khan University Hospital during the study period. Thirty-seven patients (88.1%) were females. Mean age ± standard deviation of the participants was 53.8 ± 14.7 years. Sixteen patients had secondary infertility and all were females; a majority of patients in this group had Sheehan’s syndrome (n=8) followed by empty sella syndrome (n=3), partial empty sella syndrome (n=2), idiopathic cause (n=2) and tuberculoma (n=1). Eighteen females (48.6%) reported inability to lactate.

**Conclusions:**

Non-traumatic hypopituitarism was more common in women, with Sheehan syndrome being the most common cause of hypopituitarism in our study (35.7%). Secondary hypothyroidism was the most common hormonal deficiency. The most commonly reported symptom was weakness.

## INTRODUCTION

The term hypopituitarism denotes the deficiency of one or more of the hormones of the anterior or posterior pituitary gland whereas, panhypopituitarism is defined as the loss of all the pituitary hormones and the term is often used in clinical practice to describe patients with deficiency in growth hormone (GH), gonadotropins, corticotropin, and thyrotropin. The posterior pituitary function may remain intact in these patients.^1^

Hypopituitarism is a rare disorder with an estimated incidence of 2.07- 4.2 cases per 100,000 per year and a prevalence of 37.5- 45.5 cases per 100,000 per year with no gender difference reported.^2^,^3^ It is most commonly caused by pituitary tumors, pituitary surgery or radiotherapy. Other causes include pituitary apoplexy, Sheehan’s syndrome, stroke, traumatic brain injury and subarachnoid hemorrhage. Rarely, infiltrative processes such as lymphocytic hypophysitis, sarcoidosis and hemochromatosis cause hypopituitarism.^4^ A retrospective study of United Kingdom involving 172 adults with partial or complete hypopituitarism diagnosed between 1967 and 1994 were found to have the following causes for the pituitary disease: pituitary tumor or treatment of the tumor (76%), an extra pituitary tumor (13%), unknown cause (8%), sarcoidosis (1%) and Sheehan’s syndrome (0.5%).^5^ In contrast, a study from east India reported commonest etiology of hypopituitarism to be pituitary tumors (40%), Sheehan’s syndrome (8%) and tuberculosis (3%).^6^ This reflects that Sheehan syndrome is still a common cause of hypopituitarism in developing countries.^7^,^8^ The presentation of any cause hypopituitarism includes progressive loss of pituitary hormone secretion. This results in impaired quality of life, morbidity and mortality.^9^ The sequence of hormonal loss is highly variable, however, the usual pattern shows that GH is earliest to be lost. The next hormonal axis to be affected is the gonadotropins; the Luteinizing Hormone (LH) and Follicle Stimulating Hormone (FSH), followed by adrenocorticotrophic hormone (ACTH) and finally loss of thyrotropin (TSH) secretion.^10^

Hypopituitarism is generally difficult to diagnose and the diagnosis is often delayed or missed because of lack of understanding of this condition. To facilitate early recognition, diagnosis and timely management of this condition, we aimed to study its common clinical and biochemical presentation in a tertiary setup of Pakistan, which has never been reported before in the literature.

## METHODS

### Ethics approval

This research has been conducted on the human data after institution’s ethical review committee approval (ERC number 3043-Med-ERC-14).

### Data collection

We performed a retrospective analysis of patients with hypopituitarism from January 2004 to December 2013 at the Section of Endocrinology, Department of Medicine, Aga Khan University Hospital, which is a large tertiary care center in Pakistan. It has specialized neurosurgery unit and medical records are maintained in the Health Information and Management System (HIMS) under Quality Control policy of the hospital. A list was generated by HIMS by using the search term of hypopituitarism. Patients with hypopituitarism secondary to sellar or parasellar tumors and traumatic brain injury were excluded. We reviewed the specific causes of hypopituitarism from inpatient and outpatient medical records, as documented by the caring endocrinologists, neurosurgeons and obstetricians. Information was recorded using data collection sheet and included the demographic and clinical parameters. The biochemical profile consists of assessment of anterior pituitary hormones function including ACTH, morning cortisol, TSH, free thyroxine levels, prolactin, LH, FSH, Testosterone, Estradiol, Insulin like growth factor-1 and GH. Data was collected by trained medical doctors and double checked by principal investigator.

### Statistical analysis

The statistical analysis was conducted by using the Statistical package for social science SPSS (Release 16.0 standard version, copyright © SPSS). A descriptive analysis was performed for demographic and clinical characteristics and results are presented as mean ± standard deviation for quantitative variables and numbers (percentages) for qualitative variables.

## RESULTS

### Demographic & clinical characteristics of hypopituitarism patients

During nine years, a total of 42 patients were diagnosed to have hypopituitarism other than sellar or parasellar tumors and traumatic brain injury. Thirty-seven patients (88.1%) were females. Table-I shows the demographic and clinical manifestations of these patients. Mean age ± standard deviation of the participants was 53.8 ± 14.7 years with 50% having age greater than 55 years. The median duration of experiencing symptoms was 2.7 years (interquartile range: 0.72-5.95 years). Sixteen patients had secondary infertility and all were females; a majority of patients in this group had Sheehan’s syndrome (n=8) followed by empty sella syndrome (n=3), partial empty sella syndrome (n=2), idiopathic cause (n=2) and tuberculoma (n=1). Eighteen females (48.6%) reported inability to lactate. In our sample, hypopituitarism was found to be more prevalent amongst women. Of the total sample, Sheehan’s syndrome (n=15) were all women, tuberculoma of pituitary gland were also found in women only (n=2), out of 10 patients having empty sella syndrome, seven were women, hypoplastic pituitary gland was found in two women and one man. Idiopathic cause was found in five patients and all were women. In addition, partial empty sella syndrome was found in six women and one man.

**Table I:** Demographic characteristics & clinical manifestations of patients with hypopituitarism.

Characteristics	Number of Patients (%)
*Age*	
18-35	5 (11.9)
36-55	16 (38.1)
>55	21 (50.0)
*Gender*	
Male	5 (11.9)
Female	37 (88.1)
Duration of Illness	4.4 ± 4.9 years
Decreased Libido	30 (71.4)
Decreased Body Hair	25 (59.5)
Amenorrhea/Oligomenorrhea	33 (91.6)
Diminished sense of well being	40 (95.2)
Weight loss	29 (69.0)
Lethargy	38 (90.5)
Cold Intolerance	20 (47.6)
Decreased Appetite	36 (85.7)
Constipation	12 (28.6)
Facial puffiness	7 (16.7)
Headache	13 (31.0)
Visual disturbance	3 (7.1)
Altered Level of consciousness	20 (47.6)
Seizures	6 (14.3)
Hypotension	12 (28.6)
Anemia	20 (47.6)
Hyponatremia	30 (71.4)
Arrythmias	4 (9.5)

### Etiology & Hormonal profile of hypopituitarism

**Fig. 1:**
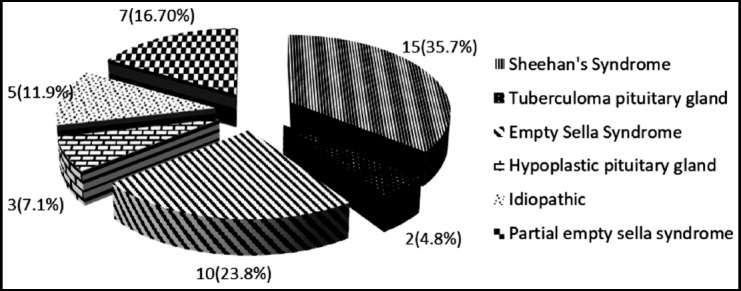
Causes of hypopituitarism in patients presenting to a tertiary care hospital, n (%).

**Fig. 2:**
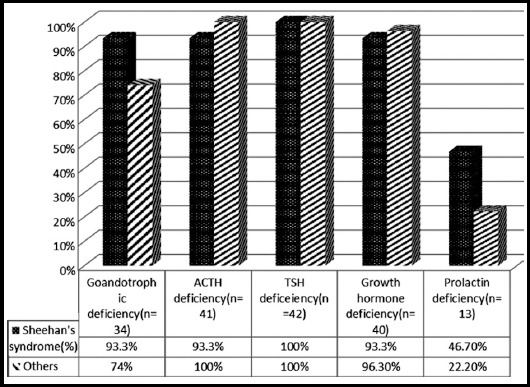
Percentage of patients with documented hormonal deficiencies in Sheehan`s Syndrome and others (n=42).

**Fig. 3:**
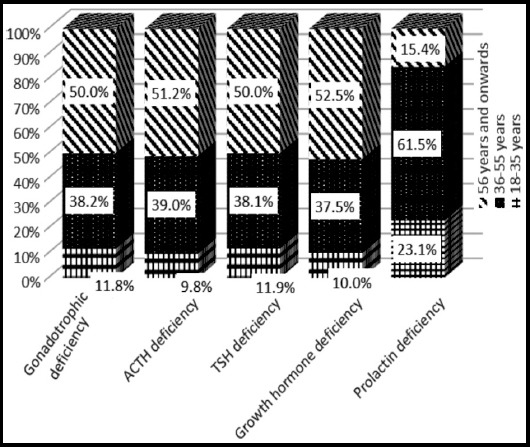
Age distribution of patients with different hormonal deficiencies (n= 42).

Fig. 1 presents the distribution of etiology of hypopituitarism in these patients. The most common etiology was Sheehan’s syndrome (35.7%). Table-II, shows comparison of Sheehan’s syndrome with other causes of hypopituitarism with no significant difference in demographic, clinical and biochemical parameters. Distribution of patients with hormone deficiencies and age groups is presented in Fig. 2 and 3.

**Table II:** Differences according to etiology of hypopituitarism.

Characteristics	Sheehan’s Syndrome N=15 (35.7%)	Others N=27 (64.3%)	P-Value
*Age groups (years)*			
18-35	3 (20.0)	2 (7.4)	0.22
36-55	7 (46.7)	9 (33.3)	
56 and above	5 (33.3)	16 (59.3)
*Gender*			
Men	0(0)	5(18.5)	0.07
Women	15(100)	22(81.5)	
Duration of Illness, mean (SD) (months)	42.8 (35.9)	58.6 (69.8)	0.42
Decreased Libido	13 (86.7)	17 (63.0)	0.10
Infertility	8 (53.3)	8 (30.8)	0.15
Decreased Body Hair	10 (66.7)	15 (55.6)	0.48
Diminished sense of well being	15 (100)	25 (92.6)	0.28
Weakness	15 (100)	26 (96.3)	0.45
Hyponatremia	9 (60)	21 (77.8)	0.22
Hypoglycemia	4 (26.7)	2 (7.4)	0.08
Hypotension	6 (40.0)	6 (22.2)	0.22
Eosinophilia	2 (13.3)	3 (11.1)	0.83
Fatigue	14 (93.3)	24 (88.9)	0.63
Weight loss	12 (80.0)	17 (63.0)	0.25
Lethargy	14 (93.3)	24 (88.9)	0.63
Cold intolerance	9 (60.0)	11 (40.7)	0.23
Decreased appetite	14 (93.3)	22 (81.5)	0.29
Constipation	5 (33.3)	7 (25.9)	0.611
Facial Puffiness	3 (20.0)	4 (14.8)	0.66
Bradycardia	0 (0)	1 (3.7)	0.45
Anemia	7 (46.7)	13 (48.1)	0.92
Headache	4 (26.7)	9 (33.3)	0.65
Visual Disturbance	0 (0)	3 (11.1)	0.18
Altered Level of consciousness	10 (66.7)	10 (37.0)	0.06
Seizures	2 (13.3)	4 (14.8)	0.89
Arrhythmia	1 (6.7)	3 (11.1)	0.63
*Body Mass Index, mean(SD)*			
Underweight (≤ 18.4)	3 (25.0)	4 (22.2)	0.52
Acceptable (18.5-22.9)	3 (25.0)	3 (16.7)	
Overweight (23-24.9)	3 (25.0)	2 (11.1)	
Obese (25 and above)	3 (25.0)	9 (50.0)	
Gonadotrophic deficiency	14 (93.3)	20 (74.1)	0.12
ACTH deficiency	14 (93.3)	27 (100)	0.17
TSH deficiency	15 (100)	27 (100)	-
Growth hormone deficiency	14 (93.3)	26 (96.3)	0.66
Prolactin Deficiency	7 (46.7)	6 (22.2)	0.10
Heart rate, mean(SD) (rates/min)	84.2 (13.1)	76.2 (9.3)	0.04
Blood pressure, systolic, mean(SD) (mmHg)	135.4 (28.2)	123.3 (22.4)	0.16
Blood pressure, diastolic, mean(SD) (mmHg)	73.4 (12.6)	72.6 (10.9)	0.83
Weight, mean(SD) (Kg)	52.5 (12.6)	59.6 (15.5)	0.17
Height, mean(SD) (cm)	153.4 (6.0)	155.8 (7.8)	0.37

†SD: Standard deviation.

## DISCUSSION

### Prevalence of hypopituitarism

A study conducted in a tertiary care center in India reports most common cause of hypopituitarism to be a non-functioning pituitary adenoma followed by Sheehan’s syndrome (27%).^11^ As we have excluded sellar and parasellar tumors, which remains the commonest cause, we found that 35.7% of hypopituitarism was attributable to Sheehan’s syndrome, which is close to a Turkish study (107 of 388 patients; 27.6%).^12^ Sheehan’s syndrome also accounted for the cause of hypopituitarism in 41% (15/22) of women in our study. This is probably because the prevalence of Sheehan syndrome is still very common in developing countries, due to inadequate availability of obstetric care. In contrast the western data reported only 0.5% cases of Sheehan’s syndrome causing hypopituitarism in one of the study.^5^ Empty sella syndrome was the next most common cause of hypopituitarism in our study population (23.8%) which is different to the 7.3% frequency reported by Chatterjee et al.^6^ We had only five men in our study, all of whom had presented with decreased libido, weakness and fatigue and decreased appetite, besides other complaints. The most common etiology of hypopituitarism was empty sella syndrome (80%). Around 11.9% cases had idiopathic hypopituitarism with almost similar figure reported by Tanriverdi et al. (10.6%).^12^ Hypopituitarism was also reported in 20% of a small subset of patients after recovery from tuberculous meningitis in a study done in a teaching hospital.^13^

### Spectrum of hormonal deficiency

Most common hormonal deficiency was found to be TSH with 100% of the study population being affected, followed by hypocortisolism secondary to ACTH deficiency (97.6%) and growth hormone deficiency (95.2%). Almost a similar study conducted in India reported the clinical spectrum of hypopituitarism with hypogonadism (97%) as the most common abnormality, followed by growth hormone deficiency (88.1% of the 42 patients tested), hypothyroidism (83.2%), hypoadrenalism (79.6%), and diabetes insipidus (13.3%).^13^

### Clinical features of hypopituitarism

Most commonly reported symptoms in our study populations was weakness (97.6%), diminished sense of wellbeing (95.2%) and lethargy (90.5%). These symptoms either reflect the manifestation of electrolytic disturbances like hyponatremia, found in about 71.4% of our study population or due to hypothyroidism and adrenal insufficiency. An interesting finding of increased hypoglycemia in Sheehan’s syndrome compared to other causes is speculative, but not significant (p=0.08) in our study. Although presentation with hypoglycemia is rare, but this is increasingly being reported to be due to an acute as well as delayed presentation of varying pituitary deficiencies, especially the hypothalamus-pituitary-adrenal axis.^14^-^18^ Our patients with Sheehan’s syndrome also had significantly higher heart rate compared to other causes of hypopituitarism (p=0.04), reflecting the hemodynamic changes associated with the chronic adaptation of cardiovascular system associated with adrenal insufficiency well reported in acute as well as chronic cases.^19^-^23^ Patients in our study were all delayed in their diagnosis. However, no studies have been done to demonstrate any head-to-head comparison regarding circulatory dynamic changes in patients with Sheehan’s and other causes of hypopituitarism, although such changes are equally represented in the later.^24^-^26^

There may be number of methodological limitations associated with a retrospective study design, as most of the patients are catered when they are extremely symptomatic. Medical record reviews are dependent on the content of the medical charts and the care with which it was recorded. However, considering these limitations, this is the only study from one tertiary health care hospital out of a population of 207.8 million people in Pakistan. We propose to do much larger studies in the future.

## CONCLUSIONS

We conclude that Sheehan’s syndrome is the most common cause of hypopituitarism in Pakistan after excluding patients of sellar and suprasellar tumors. Most common hormonal deficiency was TSH and most commonly reported symptoms were weakness. Sheehan syndrome can be prevented by provision of adequate obstetric care especially in patients with low socio-economic status.
